# Ultrasonic cartilage thickness measurement is accurate, reproducible, and reliable—validation study using contrast-enhanced micro-CT

**DOI:** 10.1186/s13018-019-1099-8

**Published:** 2019-02-27

**Authors:** Simon Damian Steppacher, Markus Simon Hanke, Corinne Andrea Zurmühle, Pascal Cyrill Haefeli, Frank Michael Klenke, Moritz Tannast

**Affiliations:** Department of Orthopaedic Surgery and Traumatology, Inselspital, Bern University Hospital, University of Bern, CH-3010 Bern, Switzerland

**Keywords:** Cartilage thickness, Ultrasonography, Micro-CT, Validation, Accuracy

## Abstract

**Background:**

Ultrasonography is a fast and patient-friendly modality to assess cartilage thickness. However, inconsistent results regarding accuracy have been reported. Therefore, we asked what are (1) the accuracy, (2) reproducibility, and (3) reliability of ultrasonographic cartilage thickness measurement using contrast-enhanced micro-CT for validation?

**Methods:**

A series of 50 cartilage–bone plugs were harvested from fresh bovine and porcine joints. Ultrasonic cartilage thickness was determined using an A-mode, 20-MHz hand-held ultrasonic probe with native (1580 m/s) and adjusted speed of sound (1696 m/s). All measurements were performed by two observers at two different occasions. Angle of insonation was controlled by tilting the device and recording minimal thickness. Retrieval of exact location for measurement was facilitated by aligning the circular design of both cartilage–bone plug and ultrasonic device. There was no soft tissue interference between cartilage surface and ultrasonic probe. Ground truth measurement was performed using micro-CT with iodine contrast agent and a voxel size of 16 μm. The mean cartilage thickness was 1.383 ± 0.402 mm (range, 0.588–2.460 mm).

**Results:**

Mean accuracy was 0.074 ± 0.061 mm (0.002–0.256 mm) for native and 0.093 ± 0.098 mm (0.000–0.401 mm) for adjusted speed of sound. Bland–Altman analysis showed no systematic error. High correlation was found for native and adjusted speed of sound with contrast-enhanced micro-CT (both *r* = 0.973; *p* < 0.001). A perfect agreement for reproducibility (intraclass correlation coefficient [ICC] 0.992 and 0.994) and reliability (ICC 0.993, 95% confidence interval 0.990–0.995) was found.

**Conclusions:**

Ultrasonic cartilage thickness measurement could be shown to be highly accurate, reliable, and reproducible. The A-mode ultrasonic cartilage thickness measurement is a fast and patient-friendly modality which can detect early joint degeneration and facilitate decision making in joint preserving surgery.

**Electronic supplementary material:**

The online version of this article (10.1186/s13018-019-1099-8) contains supplementary material, which is available to authorized users.

## Background

Early cartilage degeneration is characterized by softening and thinning. In the advanced stage, the cartilage deteriorates with partial and full thickness defects [1]. Measurement of cartilage thickness allows early and objective evaluation of joints in an early arthritic stage. In addition, outcome following disease modification, e.g., joint-preserving surgery, can be quantified by monitoring cartilage thickness. While conventional radiography or computed tomography (CT) imaging of the joint could be shown to be relatively insensitive for early arthritic changes, magnetic resonance (MR) imaging is the today’s gold standard for cartilage evaluation [2]. However, MR imaging is a costly and time-consuming imaging modality often necessitating intraarticular contrast agent for accurate evaluation of cartilage. Ultrasonographic imaging is an inexpensive, fast, and patient-friendly alternative for cartilage thickness evaluation. It offers the possibility of real-time cartilage evaluation and could potentially be applied intraoperatively during joint surgery.

There are, however, inconsistent results regarding the accuracy of ultrasonic cartilage thickness measurement in literature [3]. While some studies have shown a good accuracy for this method of cartilage thickness measurement [4–6], others did not recommend it as valid [7–10]. Inaccurate results could potentially be due to unconsidered acoustic principals of ultrasonography [3], the use of an inaccurate ground truth measurement [8, 10, 11], or soft tissue interference between the measurement probe and cartilage surface.

The current study evaluated a hand-held device for A-mode ultrasonic cartilage thickness measurement. For validation of ultrasonic cartilage thickness measurement, the results were compared to the thickness measurements using contrast-enhanced micro-CT, a method with very high spatial resolution in the micrometer range. The ex vivo evaluation was performed using fresh bovine and porcine cartilage samples. The purpose of the study was to determine if using contrast-enhanced micro-CT as the validation tool would demonstrate that A-mode ultrasound is an (1) accurate (2), reproducible, and (3) reliable method of measuring cartilage thickness. In addition, the goal was to summarize and compare the results in literature about A-mode cartilage thickness measurement and to show potential errors resulting in decreased accuracy of ultrasonic measurement.

## Methods

### Specimens

A total of 50 cylindrical cartilage–bone plugs were harvested from bovine and porcine joints. This included six bovine hips (15 samples from femoral heads), six bovine knees (11 samples from femoral condyles, four from tibial plateaus, three from patellae), five porcine shoulders (15 samples from humeral heads), and one porcine elbow (two samples from the trochlea). All joints had macroscopically intact cartilage and a closed joint capsule until cartilage–bone plugs were harvested using a 10-mm cylindrical punch. All plugs had a minimum thickness of 5 mm subchondral bone. Ultrasonic cartilage thickness measurement was performed immediately following the harvest of the plugs. Until micro-CT measurement was performed, the cartilage–bone plugs were stored in phosphate-buffered saline solution at 4 °C. All measurements were performed within maximum 18 h from slaughter.

### Ultrasonographic cartilage thickness measurement

**Fig. 1:**
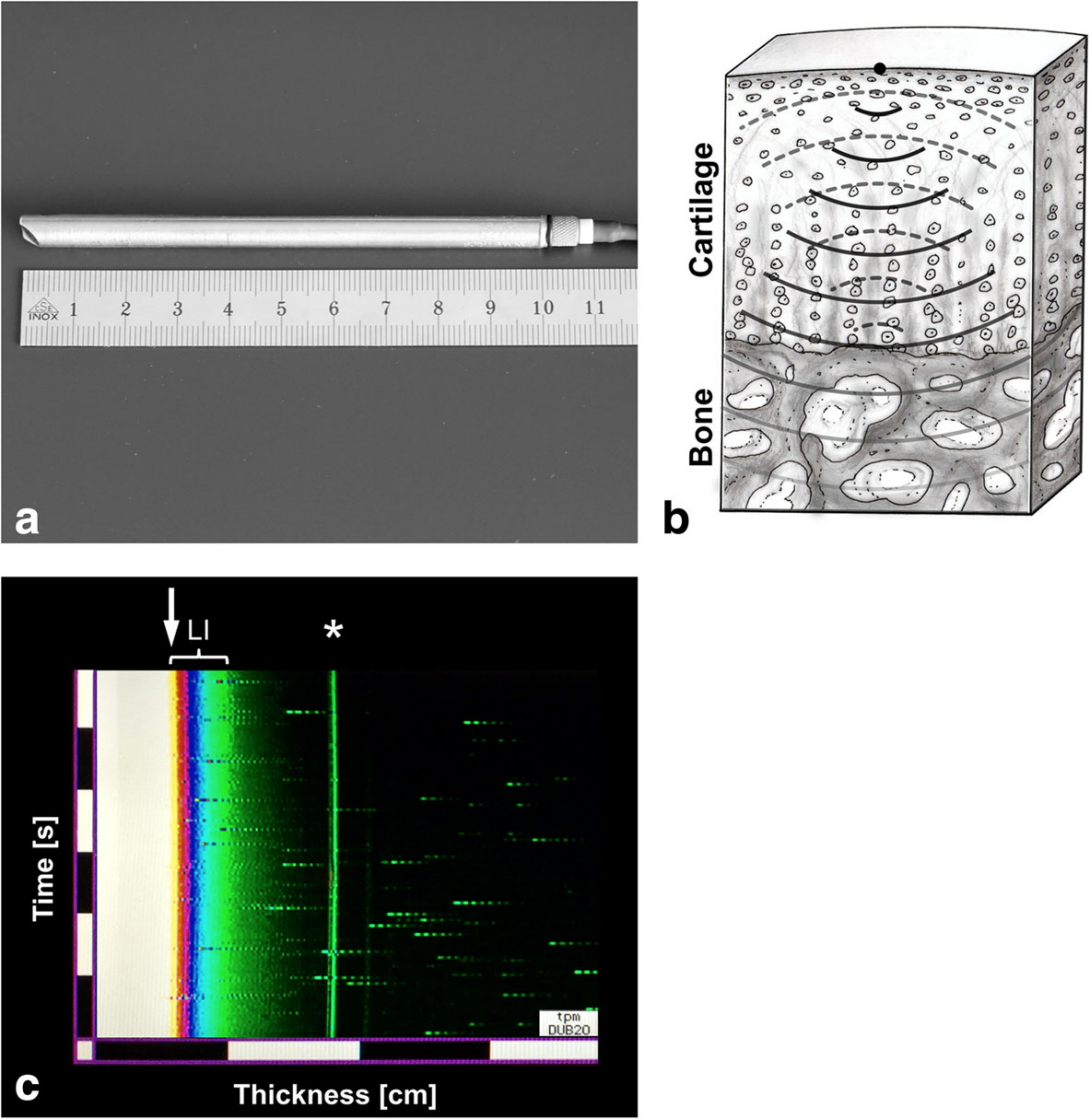
**a** A pen-like ultrasonic transducer was used (DUB micro®, Rev. 2.14a, Taberna pro medicum, Lüneburg, Germany). **b** The ultrasonic signal fades while traveling through cartilage. At the cartilage–bone border, the signal is partially reflected (dashed lines). Cartilage thickness is calculated based on the time the reflected signal needs to travel through cartilage. Therefore, thickness directly relates to speed of sound in cartilage. **c** A-mode ultrasonic image: upper cartilage border (arrow); “leading interface” (LI), an interference pattern which occurs due to the great difference in acoustic impedance of cartilage and gel; cartilage–bone border (asterisk)

Ultrasonic cartilage thickness was determined with an ultrasonic hand-held probe (DUB micro®, Rev. 2.14a, Taberna pro medicum, Lüneburg, Germany). Thickness was measured at the center of the cartilage plug, and the ultrasonic probe was manually aligned. Both the cartilage probe and the ultrasonic device had a circular design (Fig. 1), which facilitated alignment. The gap between the ultrasonic probe and the cartilage surface was filled with ultrasound transmission gel (Aquasonic® 100, Parker Laboratories, Fairfield NJ, USA). An average layer of transmission gel of 0.745 ± 0.018 mm (range, 0.704 to 0.792 mm) was used. The speed of sound of the ultrasonic probe was 1580 m/s. The 20-MHz echoes were plotted on a screen as a function of depth and time (A-mode sonography). Cartilage thickness was measured between the first and second reflection (Fig. 1). The band-shaped interference following the first reflection, also known as the “leading interface” [3], is a part of the cartilage and was included in the thickness measurement (Fig. 1). This “leading interface” occurs due to the considerable difference in acoustic impedance of the transmission gel and cartilage [3]. The angle of insonation has a significant influence on cartilage thickness measurement. Deviation from true orthogonal insonation will result in increased cartilage thickness measurement. Therefore, the ultrasonic probe was tilted manually during continuous measurement, and the smallest plotted cartilage thickness was recorded (Fig. 1). Cartilage thickness was measured with native speed of sound of the ultrasonic probe with 1580 m/s. An optimal speed of sound of 1696 m/s for cartilage thickness measurement has been recommended in literature [3, 9, 12]. Therefore, cartilage thickness evaluated with native speed of sound was multiplied by the factor 1.07 (1696/1580) for calculation of thickness with optimal speed of sound. The thickness measurements were performed by two observers and twice for each cartilage–bone plug by each observer. The average cartilage thickness of all 200 ultrasonic measurements ranged from 0.595 to 2.464 mm (mean 1.372 ± 0.412 mm).

### Contrast-enhanced micro-CT

**Fig. 2:**
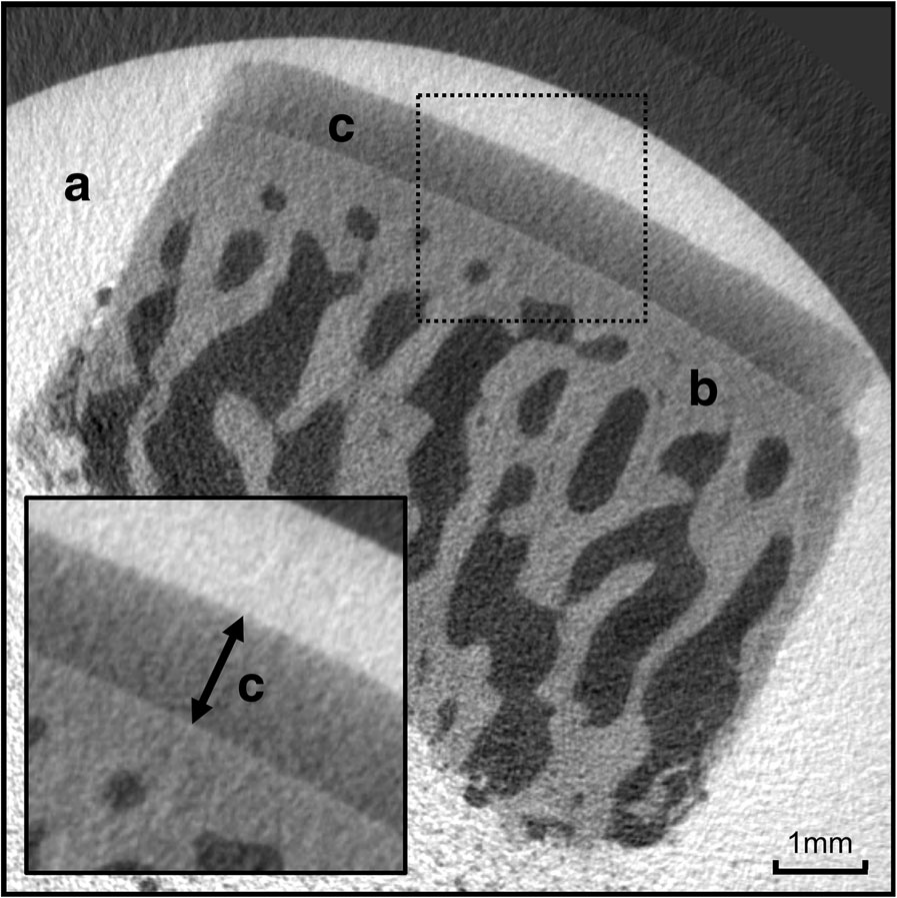
For validation of ultrasonic cartilage thickness measurement we used the micro-CT with iodine contrast agent (**a**) for comparison. For micro-CT measurements the cartilage-bone samples were harvested with a punch biopsy and embedded in a radiolucent tube. While the lower limit of the cartilage (**c**) adjacent to the bone (**b**) was clearly visible in the micro-CT, detection of the upper limit of the cartilage was enhanced by iodine contrast agent (**a**). This allowed exact determination of cartilage thickness

Ground truth measurement of cartilage thickness was done using contrast-enhanced micro-CT (Scanco Medical μCT 40, version 6.1, Brüttisellen, Switzerland). The scans were performed with the following parameters: voxel size of 16 μm, maximum voltage of 70 kVp, and electric current of 114 μA. Each cartilage–bone sample was embedded in a radiolucent sampling tube with 15-mm diameter (Fig. 2). To restrict movements of the cartilage–bone plugs during micro-CT scan, a sponge was inserted into the tube. While the bone–cartilage interface is clearly identifiable using native CT, the cartilage–air interface of the cartilage is difficult to be distinguished. Therefore, the cartilage–bone plugs were embedded in a solution with a 1:1 ratio of iodine contrast agent (Iopamidol, 300 mg/ml, Bracco Suisse SA, Manno, Switzerland) and phosphate-buffered saline (Fig. 2). Scan volume was determined on the scout view with a constant 15-mm diameter and individual height ranging from 5.9 to 15.5 mm. Cartilage thickness was measured using a DICOM viewer (Osirix, version 5.8, Geneva, Switzerland). Multiplanar reconstruction was used to obtain a cross-section perpendicular to the cartilage surface, and the cartilage thickness was measured at the center of the cartilage–bone plug (Fig. 2). The mean cartilage thickness measured using micro-CT was 1.383 ± 0.402 mm (range, 0.588–2.460 mm; see Additional file 1 named “Data validation ultrasonic cartilage thickness measurement.xls”).

### Validation

Accuracy of ultrasonic cartilage thickness measurement was evaluated by calculating the difference between the ultrasonic and the micro-CT measurements of cartilage thickness. In addition, the error was calculated as the quotient of the difference of cartilage thickness using the two modalities and the thickness evaluated using micro-CT. The linear relationship between the ultrasonic and micro-CT techniques for cartilage thickness was calculated. All measurements were made for both native and adjusted speed of sound in cartilage.

Reproducibility was evaluated by comparing the ultrasonic measurements performed at two occasions. Reliability was evaluated by comparing the measurements of the two observers. Reproducibility and reliability measurements were performed with native speed of sound in cartilage.

### Statistical analysis

Accuracy was calculated as the absolute difference of the ultrasonic and micro-CT-based cartilage thickness measurements. Differences in accuracy or error between ultrasonic measurement with native and adjusted speed of sound were detected using the independent *t* test. To detect a systematical error of the ultrasonic measurements, the Bland–Altman analysis [13] was calculated by plotting the difference between the two measurement techniques against their average. Correlation between the two measurements techniques was evaluated with the Pearson correlation coefficient. The Pearson correlation coefficient was graded as *r* < 0.2 for very weak, 0.20–0.39 for weak, 0.40–0.59 for moderate, 0.60–0.79 for strong, and ≥ 0.8 for very strong correlation [14]. The ICC was used for calculation of reproducibility and reliability and was graded as ICC < 0.20 for slight agreement, 0.21–0.40 for fair, 0.41–0.60 for moderate, 0.61–0.80 for substantial, and > 0.80 for almost perfect agreement [15]. The level of significance was set at 0.05.

## Results

**Fig. 3:**
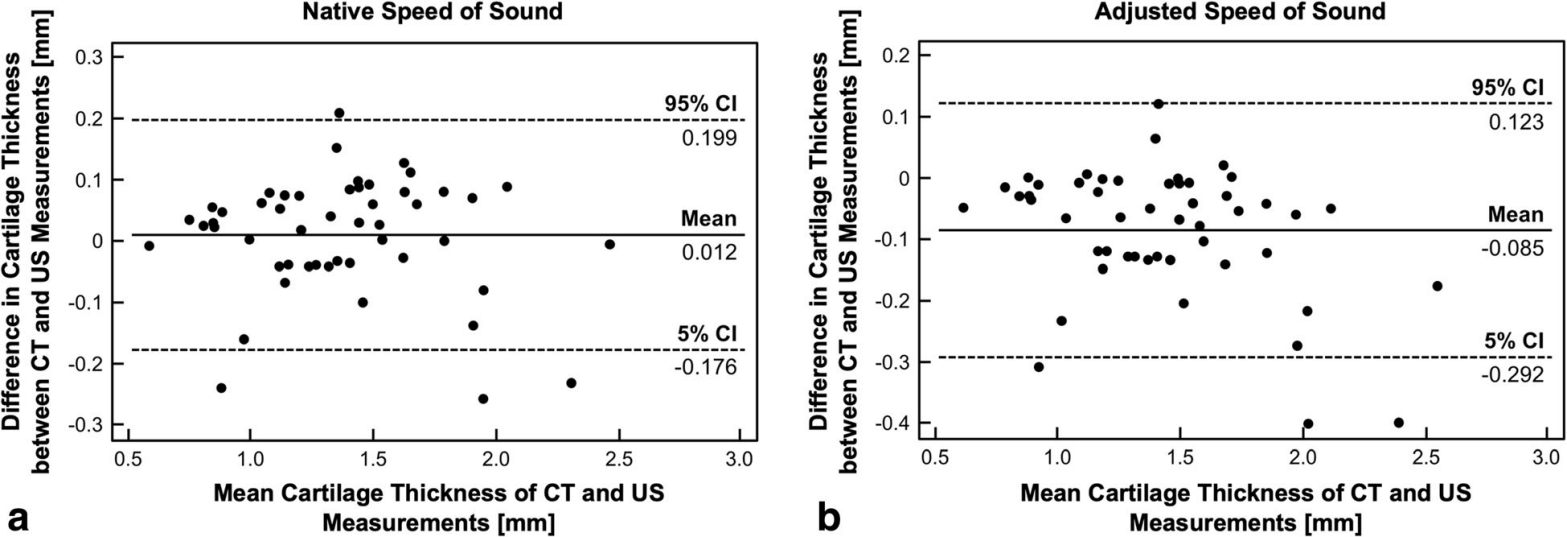
The Bland–Altman analysis [13] was performed by plotting the difference between the two measurement techniques against their average. The analysis showed that the means of the measurement pairs were spread evenly and randomly, and therefore, no systematic error existed for **a** native (1580 m/s) and **b** adjusted speed of sound (1696 m/s) in cartilage. CT = computed tomography, US = ultrasonography, CI = confidence interval

**Fig. 4:**
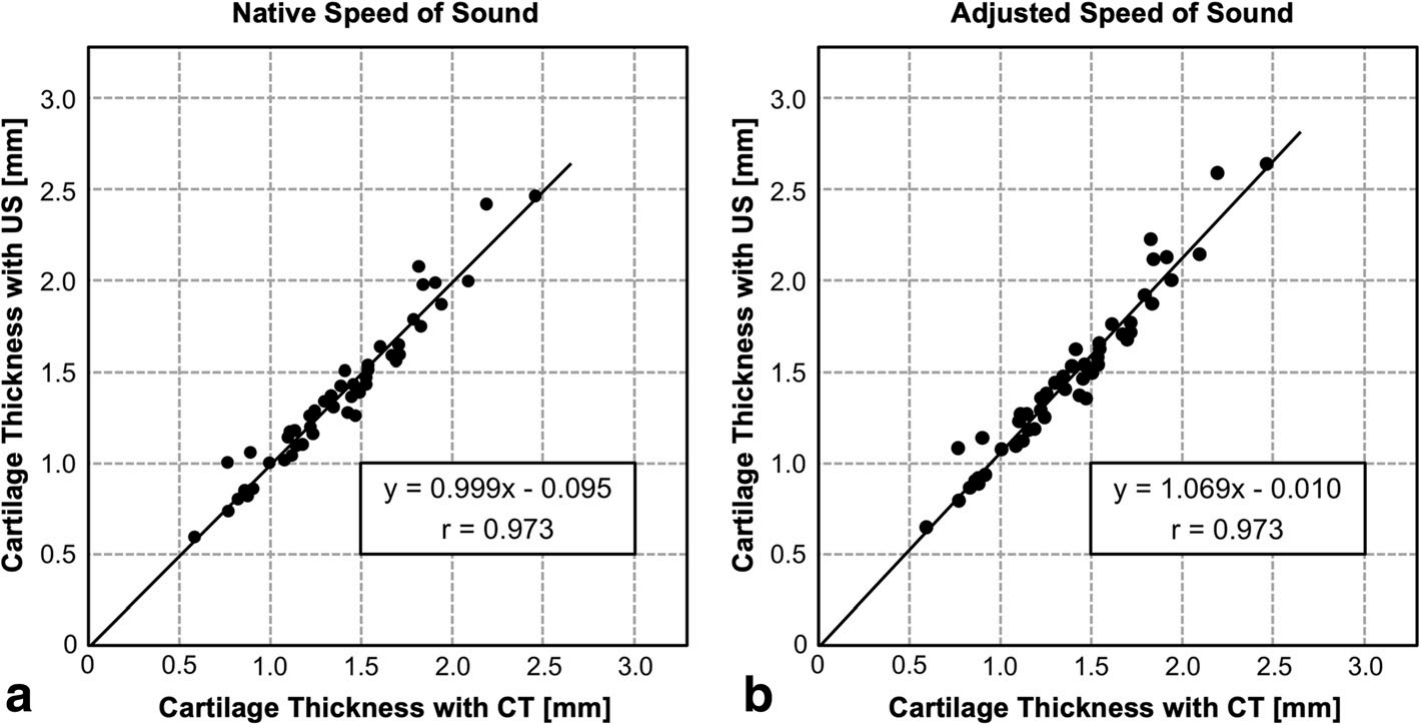
Correlation of micro-CT measurement of cartilage thickness with ultrasonic measurement **a** with native (1580 m/s) or **b** adjusted speed of sound (1696 m/s). A strong correlation between ultrasonic and micro-CT measurement was found for both native and adjusted speed of sound (both *p* < 0.001 and *r* = 0.973). CT = computed tomography, US = ultrasonograph

For the ultrasonic measurement with native speed of sound, the mean accuracy was 0.074 ± 0.061 mm (0.002–0.256 mm) with a corresponding mean error of 6% ± 5% (0–31%). For adjusted speed of sound, the mean accuracy was 0.093 ± 0.098 mm (0.000–0.401 mm) with a corresponding mean error of 7% ± 8% (0–40%). No difference existed for accuracy or error between the measurements using native or adjusted speed of sound (*p* = 0.237 and *p* = 0.289, respectively). The Bland–Altman analysis showed that the mean of the measurement pairs was spread evenly and randomly with no evidence for a systematic error for both the ultrasonic measurements with native or adjusted speed of sound (Fig. 3). A very strong linear correlation was found between the contrast-enhanced micro-CT measurement and the ultrasonic measurement using native or adjusted speed of sound (*p* < 0.001; *r* = 0.973 for both; Fig. 4).

An almost perfect agreement for both reproducibility (ICC of 0.992 and 0.994) and reliability (ICC 0.993, 95% confidence interval of 0.990–0.995) was found (Table 1).

**Table 1:** Results of reproducibility and reliability

Parameter	ICC intraobserver 1	ICC intraobserver 2	ICC interobserver
Cartilage thickness	0.992 (0.986–0.996)	0.994 (0.990–0.997)	0.993 (0.990–0.995)

Values are expressed as mean with 95% confidence interval in parentheses

*ICC* intraclass correlation coefficient

## Discussion

Ultrasonic measurement of cartilage thickness is an inexpensive, radiation-free, and patient-friendly alternative to MRI. It offers the possibility of real-time imaging which could also be applied intraoperatively, e.g., for topographical cartilage thickness assessment in joint-preserving surgery. Inconsistent results regarding accuracy for ultrasonic thickness measurement have been reported (Table 2). Several factors need to be controlled to minimize error for validation of ultrasonic cartilage thickness measurement including the angle of insonation, retrieval of location of measurement, speed of sound in cartilage, and handling of cartilage samples. In addition, ground truth measurement needs to be precise and error-free. We validated a pen-like ultrasonic device for cartilage thickness measurement. Contrast-enhanced micro-CT measurement of cartilage thickness was used as ground truth measurement for validation with an iodine contrast agent and multiplanar reconstruction. We could show that ultrasonic assessment of cartilage thickness is very accurate (mean accuracy of 0.074 mm [6%]) and has no systematical error (Fig. 3). We found a very strong correlation with micro-CT measurement of cartilage thickness (Fig. 4), and we could show an almost perfect agreement for both reproducibility and reliability (Table 1). No improvement was found for the adjusted speed of sound in cartilage (Figs. 3 and 4).

**Table 2:** Selected literature on accuracy of ultrasonic cartilage thickness measurement

Author, year	Joint	Species	Setup	*n*	Ground truth	Ultrasonic measurement	Accuracy
Aisen et al., 1984 [21]	Knee	Bovine	Cadaver ^a^	3	Ruler	B-mode, 7.5 MHz, 1750 m/s speed of sound, immersed mineral oil bad	Mean difference ranging from 0.1 to 0.2 mm (cartilage thickness from 1.0 to 4.2 mm)
Modest et al., 1989 [25]	Hip	Human	Cadaver^a^	24	Microscope Thawed cadaver, toluidine O staining	A-mode, 7.5 MHz, 1760 m/s speed of sound, thawed plugs	Comparison of the average of 5 ultrasonic with 12 microscopic measurements; 88% of the ultrasonic values within one standard deviation of the microscopic measurements
Martino et al., 1993 [22]	Knee	Human	In vivo^b^	18	Microscope Fixed in 90% ethanol, resection following TKA	B-mode, 7.5 MHz, fixed in 90% ethanol, transcutaneous	Significant difference of 0.3 mm for the minimum cartilage difference, no significant difference of 0.2 mm for the maximum cartilage thickness
Jurvelin et al., 1995 [4]	Knee	Canine and bovine	Cadaver^a^	45	Microscope/needle probe Temporarily stored in 4 °C Ringer solution	A-mode, 10 MHz, temporarily stored at 4 °C Ringer solution, 1760 m/s speed of sound	A strong correlation was found between ultrasonic and microscopic measurement (*r* = 0.91) or needle probe measurement (*p* = 0.90). The mean difference between microscopic and ultrasonic measurement was 0.03 ± 0.16 mm
Ostergaard et al., 1995 [18]	Knee	Human	In vivo^b^	39	MRI 1.5 Tesla, 3D fast imaging by steady-state precession, slice thickness of 1.56 mm	B-mode, 7.5 MHz, 1750 m/s speed of sound, transcutaneous	Mean difference of 0.2 mm (95% confidence interval − 1.4–1.0 mm) and a strong correlation with *r* = 0.82
Chérin et al., 2001 [19]	Patella	Murine	Cadaver^a^	48	Microscope Decalcified and fixated, hematoxylin–eosin–safran staining	B-mode, 50 MHz, measurement within minutes of sacrifice in 0.9% saline solution, 1600 m/s speed of sound	A strong correlation with *r* = 0.8923 (least square linear fit *y* = 0.6589*x* + 79.758) was found
Töyräs et al., 2002 [20]	Patella	Bovine	Cadaver^a^	60	Needle probe Plugs obtained within few hours after slaughter	A-mode, 22 MHz, 1654 m/s speed of sound. Plugs obtained within few hours after slaughter	Mean difference of 0.02 ± 0.99 mm with a strong correlation of *r* = 0.943.
Pellaumail et al., 2002 [26]	Patella	Murine	Cadaver^a^	16	Microscope Decalcified, paraffin fixated and stained with toluidine blue	B-mode, 50 MHz, measurement within minutes of sacrifice in 0.9% saline solution, 1600 m/s speed of sound	Difference ranging from 0.09 to 0.79 mm depending on age and cartilage degeneration
Tarhan et al., 2003 [8]	Knee	Human	In vivo^b^	206	MRI 0.23 Tesla, proton density and T2-weighted images, 4.5 mm slice thickness with 0.5 mm interslice gap	B-mode, 5–10 MHz, transcutaneous	Correlation coefficient *r* ranging from 0.38 to 0.74. No difference between non-arthritic (*r* ranging from 0.38 to 0.74) and arthritic knee joints (0.44 to 0.61).
Mathiesen et al., 2004 [27]	Knee	Human	Cadaver^b^	24	Caliper gauge Thawed at room temperature	B-mode, 10 MHz, thawed at room temperature	Mean difference was 0.0 ± 0.4 mm.
Yoon et al., 2008 [10]	Knee	Human	In vivo^b^	102	MRI 1.5 Tesla, fat-suppressed spoiled gradient echo images, 1.5-mm slice thickness	B-mode, 12.5 MHz, transcutaneous	Correlation coefficient *r* for the minimal and maximal cartilage thickness in arthritic knees ranged from 0.568 to 0.844 for the medial epicondyle and from 0.314 to 0.507 for the lateral epicondyle
Naredo et al., 2009 [24]	Knee	Human	Cadaver^b^	24	Stereoscopic magnifying loupe Fixed in formalin solution	B-mode, 14 MHz, thawed knees, transcutaneous	Agreement of the two methods was assessed using the ICC (intraclass correlation coefficient). The ICC was 0.719 for the medial epicondyle, 0.285 for the lateral epicondyle, and 0.267 for the intercondyle region. Mean difference ranged from 0.266 to 0.326 mm.
Aula et al., 2010 [17]	Knee	Bovine	Cadaver^a^	10	Contrast agent CT Slice thickness of 2.3 mm, pixel size of 0.2 × 0.2 mm^2^, thawed and stored in phosphate-buffered saline	A-mode, 30 MHz, thawed and stored in phosphate-buffered saline	Correlation coefficient *r* was 0.84.
Spannow et al., 2011 [28]	Knee, ankle, wrist, finger	Human	In vivo^b^	143	MRI 1.5 Tesla, fat-suppressed T1, slice thickness 1.4–1.6 mm, voxel size 0.5 × 0.8 × 1.6 mm	B-mode, 6-14 MHz, transcutaneous	Mean difference ranging from 0.08 to 0.48 mm.
Ohashi et al., 2012 [5]	Knee	Porcine	In vitro^a^	62	Microscope Thawed cadaver	Conventional B-mode and real-time spatial compound B-mode, 10 MHz, 1488 and 1709 m/s speed of sound, thawed, stored in saline solution	Correlation coefficient *r* was 0.961 for conventional and 0.976 for real-time spatial sonography. The mean difference was 0.0073 ± 0.171 for conventional and 0.0139 ± 0.131 for real-time spatial sonography
Ohashi et al., 2012 [6]	Knee	Human	In vivo^b^	505	MRI 3 Tesla, double echo steady state sequence, slice thickness 0.29 mm, in plane resolution 0.31 mm × 0.31 mm	B-mode, 10 MHz, transcutaneous	Correlation coefficient *r* was 0.976 for non-arthritic and 0.964 for arthritic knees. The mean difference was 0.085 ± 0.405 mm for non-arthritic and 0.154 ± 0.413 mm for arthritic knees
Mandl et al., 2015 [7]	Finger	Human	Cadaver^b^	19	Calibrated photos Frozen cadavers	B-mode, 7-15 MHz, 1696 m/s speed of sound	Correlation coefficient *r* was 0.509 (*R*^2^ = 0.2595)
Pradsgaard et al., 2015 [11]	Knee	Human	In vivo^b^	69	MRI 1.5 Tesla, gradient fat saturation	B-mode, 6–14 MHz, transcutaneous, 1696 m/s speed of sound	Correlation coefficient rho was 0.86 for the medial condyle, 0.71 for the lateral condyle, and 0.70 for the intercondylar region. The mean difference was 0.31 mm for the medial condyle, 0.23 mm for the lateral condyle, and 0.33 mm for the intercondylar region
Current study	Hip, knee, and shoulder	Bovine and porcine	Cadaver^a^	50	Contrast agent micro-CT Voxel size/slice thickness of 0.016 mm, stored in phosphate-buffered saline solution for max. 18 h, iodine contrast agent	A-mode, 20 MHz, 1580 m/s and 1696 m/s speed of sound, non-frozen, evaluated within few hours of sacrifice	A strong correlation with a correlation coefficient of *r* = 0.973 was found. The mean difference was 0.074 ± 0.061 mm (0.002–0.256 mm)

*n* number of measurements, *TKA* total knee arthroplasty, *MRI* magnetic resonance imaging, *CT* computer tomography

^a^Ultrasonic measurements were performed with the probe directly on cartilage surface without interfering soft tissue

^b^Ultrasonic measurements were performed with soft tissue between the probe and cartilage potentially resulting in increased measurement error

The high resolution of ultrasonography makes it an optimal imaging modality for cartilage. However, several basic acoustic principals of ultrasonography must be taken into account for correct cartilage thickness measurement [3]. First, the borders of cartilage have to be detected correctly. At the upper cartilage border, a typical interference pattern occurs due to the great difference in acoustic impedance of cartilage and the gel (Fig. 1). This interference pattern, also called the “leading interface,” is part of the cartilage and must be included for accurate cartilage thickness measurement [3]. Second, the angle of insonation needs to be controlled. The true thickness is measured with cartilage insonated orthogonally. With a 10° and 20° error, the measured cartilage thickness is increased by 1.5% and 6.4%, respectively. The angle of insonation was controlled by manually tilting the probe and recording the thinnest cartilage thickness. Third, for evaluation of accuracy, it is mandatory to find the same location of thickness measurement with both measurement techniques (ultrasonography and micro-CT). A circular design of the cartilage samples was chosen, and the center was defined as the location of measurement. The pen-like ultrasonic probe also had a circular design which simplified manual alignment with the cartilage probe. For the micro-CT measurements, the center was defined with a digital ruler after multiplanar reconstruction to achieve orthogonal slices. Fourth, cartilage thickness directly relates on the speed of sound (Fig. 1). Most studies recommend an increased speed of sound for cartilage evaluation (average of 1696 m/s) and reported an underestimated thickness using native speed of sound [3, 9, 12]. We calculated cartilage thickness using both native and adjusted speed of sound. However, some uncertainty exists in literature regarding the correct speed of sound in cartilage, and a wide range of optimal speed from 1419 to 2428 m/s has been reported [9, 12, 16]. These discrepancies in speed were related to the different anatomical sites, cartilage degeneration, and the inhomogeneous structural components of cartilage [9, 12, 16].

Besides the acoustic principals of ultrasonography, the handling of the cartilage samples can affect accuracy. We performed both ultrasonic and micro-CT measurements within a maximum of 18 h from slaughter, and the joints were not opened before measurement. No fixation of cartilage samples was used since fixation can affect cartilage thickness by swelling or shrinking. The ground truth measurement should be precise and error-free. We used contrast-enhanced micro-CT measurement as the ground truth for validation. The micro-CT offers a very high spatial resolution up to 16 μm. The iodine contrast agent enhanced the detection of the cartilage border. The multiplanar reconstruction allowed reconstruction of orthogonal slices to measure true cartilage thickness. All these factors were controlled as good as possible to reduce potential sources of error. All cartilage samples showed macroscopically intact cartilage, and therefore, no statement can be made about the accuracy of ultrasonographic thickness measurement of cartilage with degenerative changes. In vivo measurements of cartilage thickness usually include soft tissue between the ultrasonic probe and the cartilage surface potentially resulting in increased error of cartilage thickness measurement. The setup in the current study did not include the validation with soft tissue interference. Therefore, the results of the current study do not allow to draw a conclusion on transcutaneous cartilage thickness measurement.

A high mean accuracy of ultrasonic cartilage thickness measurement of 0.074 mm (6% mean error) and 0.093 mm (7% mean error) was revealed in the current study for native and adjusted speed of sound, respectively. In addition, an almost perfect agreement between the ultrasonic and micro-CT measurements with a correlation coefficient of *r* = 0.973 was found (Fig. 4). In literature, two different methods of ground truth measurement were used including imaging (MRI or CT) or visual measurement (microscope, needle probe). For both methods of ground truth measurement, different results for accuracy have been found previously (Table 2). Some studies using imaging [6, 17, 18] or visual measurements [4, 5, 19, 20] for ground truth measurement in knee joints found an almost perfect correlation (correlation coefficient ranging from 0.82 to 0.98). In contrast, some in vivo MRI-based studies did not find satisfactory agreement with ultrasonic measurement (correlation coefficient ranging from 0.38 to 0.71) [8, 10, 11]. This might be due to inadequate spatial resolution with a slice thickness ranging from 1.5 to 4.5 mm [8, 10], no or an insufficient guidance to retrieve the exact same location of cartilage thickness measurement [8, 10, 11], and no control of the angle of insonation [8, 10]. The moderate correlation coefficient of *r* = 0.509 in another study might be due to the use of coarse ground truth measurement with calibrated photos of cross-sections of metacarpal cartilage [7]. The mean difference between ultrasonic cartilage thickness measurement and ground truth measurement published in literature ranged from 0.01 to 0.33 mm (Table 2). In the studies reporting a mean difference exceeding 0.1 mm, the inferior result of accuracy might be due to the use of a simple ruler as ground truth [21, 22], historic ultrasonic techniques [21, 22], a MRI slice thickness of 1.56 mm [18], or transcutaneous measurements with soft-tissue interference resulting in increased error of measurements [11, 22].

We found an almost perfect agreement for reproducibility and reliability with a mean ICC exceeding 0.99 (Table 1). In literature, the mean ICC for reproducibility and reliability ranged from 0.70 to 0.99 and 0.62 to 0.99, respectively [7, 10, 23, 24]. Direct insonation of the cartilage without interfering soft tissue might have decreased potential sources of error in the current study. In addition, the circular design of both the cartilage–bone plugs and the ultrasonic probe simplified retrieval of the same location for cartilage thickness measurement. The angle of insonation was controlled by manually tilting the probe and recording the thinnest cartilage thickness. These factors might also have improved the reproducibility and reliability in the current study. In literature, results for reproducibility and reliability of ultrasonic thickness measurement were based on transcutaneous measurements of cartilage in the knee and metacarpal joints with soft-tissue interference [7, 10, 23, 24]. However, this may hinder exact retrieval of the same location of cartilage thickness measurement and complicate orthogonal insonation of the cartilage.

Despite the efforts to control factors negatively affecting accuracy, several possible sources for errors in accuracy exist. First, the angle of insonation and retrieval of location of measurement were controlled manually only. Second, a maximum of 18 h existed between harvesting of cartilage sample and micro-CT measurement. During this time, the cartilage was stored in phosphate-buffered saline solution at 4 °C. This could potentially have resulted in dehydration or swelling of the cartilage affecting thickness measurement. However, since the samples were only stored for a few hours in this isotonic solution, this should not have jeopardized our results significantly. Third, adjustment of speed of sound did not improve accuracy. In contrast, with native speed of sound (1580 m/s), a slightly decreased mean error of 6% was found compared to 7% using adjusted speed of 1696 m/s. This might be due to the use of an average layer off transmission gel of 0.745 mm (54% of total thickness) mainly consisting of water (Fig. 1). Water has a lower speed of sound of 1480 m/s [3]. Thus, the use of transmission gel may have decreased the average speed of sound and affecting accuracy measurement.

## Conclusion

We could show that cartilage thickness can be assessed very accurately, reproducibly, and reliably using A-mode ultrasonography. We tried to control factors adversely affecting measurement of accuracy. This included the use of fresh cartilage samples, ultrasonic and micro-CT measurements within hours of slaughter, control of the angle of insonation, and retrieval of exact location of cartilage thickness measurement. In addition, we used contrast-enhanced micro-CT measurements with multiplanar reconstruction for the true cartilage thickness measurement. No difference in accuracy was found for adjustment of higher speed of sound in cartilage. We compared the literature on ultrasonic cartilage thickness measurement (Table 2) and compared potential factors resulting in decreased accuracy for the reported measurements. Ultrasonic measurement offers the advantage of a fast, patient-friendly, and relatively inexpensive cartilage thickness assessment. It can also be applied intraoperatively for topographical cartilage thickness evaluation. Due to the lack of radiation exposure, this method seems suitable for consecutive monitoring of cartilage thickness following disease modification, e.g., joint-preserving surgery.

## Additional file

Additional file 1: The raw data of the current study for the ultrasonic and CT measurements of cartilage thickness were included as an additional file in the file. Data validation ultrasonic cartilage thickness measurement. (XLSX 10 kb)

## Abbreviations

CT: Computer tomography
DICOM: Digital imaging and communications in medicine
MR: Magnetic resonance
